# Can Ecological Momentary Assessments Be Used as Daily Markers of Personality Traits?

**DOI:** 10.1093/geroni/igab046.963

**Published:** 2021-12-17

**Authors:** Giancarlo Pasquini, Andreas Neubauer, Nicholas Eaton, Sean Clouston, Eileen Graham, Daniel Mroczek, Stacey Scott

**Affiliations:** 1 Stony Brook University, Stony Brook, New York, United States; 2 Leibniz Institute for Research and Information in Education, Frankfurt, Mecklenburg-Vorpommern, Germany; 3 Renaissance School of Medicine, Stony Brook University, Renaissance School of Medicine, Stony Brook University, New York, United States; 4 Northwestern University, Chicago, Illinois, United States

## Abstract

This study hypothesized that select ecological momentary assessment (EMA) survey items are sensitive to day-to-day fluctuations in personality traits Extraversion (E) and Neuroticism (N). As part of the Einstein Aging Study, 312 older adults (Mage=76.96 years, SD=4.85 years, range=70-90 years) completed up to 5 EMA surveys per day for 16 days and a Big Five trait personality measure. Parallel two-factor multilevel confirmatory factor analyses were conducted for E (Daily-E; Trait-E) and N (Daily-N; Trait-N). The E model showed good fit (CFI=.95; TLI=.94; RMSEA=.02) and a significant correlation of .20 between Daily-E and Trait-E factors. The N model showed poor fit (CFI=.68; TLI=.61; RMSEA=.06). Results suggest EMA items can be used as daily markers of Extraversion, yet results are unclear for Neuroticism due to poor model fit. Daily markers of Extraversion can be used to detect fluctuations in personality traits across days that may predict long-term personality change.

